# Supplementary material for: All-Atom Molecular Dynamics Simulations of the Temperature Response of Poly(glycidyl ether)s with Oligooxyethylene Side Chains Terminated with Alkyl Groups
Source: Nanomaterials (Basel). 2023 May 12;13(10):1628. doi: 10.3390/nano13101628 (PMC10220528; doi:10.3390/nano13101628)
Supplement: Supplementary file 1 [file nanomaterials-13-01628-s001.zip › nanomaterials-2332452-supplementary.pdf]

Supplementary Materials

All-Atom Molecular Dynamics Simulations of the Temperature Response of Poly(glycidyl Ether)s with Oligooxyethylene Side Chains Terminated with Alkyl Groups

Erika Terada [1], Takuya Isono [1,2], Toshifumi Satoh [1,2], Takuya Yamamoto [1,2], Toyoji Kakuchi [3] a nd Shinichiro Sato [1,2,*]

[1] Graduate School of Chemical Science and Engineering, Hokkaido University, Sapporo 060-8628, Japan; erika_terada@eis.hokudai.ac.jp (E.T.)
[2] Faculty of Engineering, Hokkaido University, Sapporo 060-8628, Japan
[3] Research Center for Polymer Materials, School of Materials Science and Engineering, Changchun University of Science and Technology, Weixing Road 7989, Changchun 130012, China
* Correspondence: s-sato@eng.hokudai.ac.jp

Table of Contents

1. Additional Tables

2. Additional Figures

1. Additional Tables

Table S1. Total number of water molecules and box length.

Sample ID	Total Number of Water Molecules	Box Length<br>Length, Width, Height (Å)
poly(MeGE)$_{2.5k}$	2321	44.7, 43.5, 52.7
poly(EtGE)$_{2.5k}$	2015	44.2, 41.9, 49.0
poly(MeEOGE)$_{2.5k}$	2012	42.1, 43.8, 49.0
poly(EtEOGE)$_{2.5k}$	2184	45.6, 47.1, 45.0
poly(MeEO2GE)$_{2.5k}$	2297	44.5, 47.9, 47.0
poly(EtEO$_2$GE)$_{2.5k}$	2287	46.6, 44.4, 48.3
poly(MeGE)$_{5k}$	3182	49.9, 53.0, 52.7
poly(EtGE)$_{5k}$	3488	58.3, 52.9, 49.0
poly(MeEOGE)$_{5k}$	2471	48.8, 44.4, 50.1
poly(EtEOGE)$_{5k}$	2423	47.7, 47.1, 48.0
poly(MeEO2GE)$_{5k}$	4213	46.7, 66.1, 56.4
poly(EtEO$_2$GE)$_{5k}$	3876	54.8, 51.6, 58.0

Table S2. Calculated $\bar{L}/L_{\max}$ of poly(EtEOGE) for molecular weights of 1250, 2500, and 5000.

Polymer ID	Temperature (K)	$\bar{L}$ (Å)	$\bar{L}/L_{\max}$	$1/\sqrt{N}$	SD	CV
poly(EtEOGE)$_{1.25k}$	278	13.56	0.41	0.35	0.12	0.30
	300	10.93	0.33	0.35	0.12	0.37
	323	11.51	0.35	0.35	0.11	0.32
	343	12.98	0.40	0.35	0.15	0.37
	368	13.41	0.41	0.35	0.14	0.35
poly(EtEOGE)$_{2.5k}$	278	16.39	0.25	0.24	0.05	0.20
	300	15.29	0.23	0.24	0.08	0.35
	323	13.05	0.20	0.24	0.07	0.34
	343	14.98	0.23	0.24	0.08	0.35
	368	15.94	0.24	0.24	0.09	0.35
poly(EtEOGE)$_{5k}$	278	27.56	0.22	0.17	0.05	0.23
	300	17.11	0.14	0.17	0.04	0.31
	323	14.82	0.12	0.17	0.04	0.36
	343	18.33	0.14	0.17	0.06	0.44
	368	18.47	0.15	0.17	0.05	0.32

Table S3. Calculated side chain lengths at each temperature.

Polymer ID	Temperature (K)	Side Chain Lengths (Å)	SD	CV
poly(MeGE)$_{2.5k}$	278	3.70	0.04	0.010
	300	3.70	0.04	0.010
	323	3.69	0.04	0.012
	343	3.67	0.04	0.012
	368	3.66	0.04	0.012
poly(EtGE)$_{2.5k}$	278	4.74	0.04	0.009
	300	4.72	0.04	0.009
	323	4.70	0.05	0.010
	343	4.69	0.05	0.010
	368	4.68	0.05	0.011
poly(MeEOGE)$_{2.5k}$	278	6.30	0.11	0.018
	300	6.24	0.12	0.020
	323	6.20	0.14	0.022
	343	6.16	0.14	0.023
	368	6.13	0.15	0.025
poly(EtEOGE)$_{2.5k}$	278	7.06	0.24	0.034
	300	7.01	0.27	0.038
	323	7.08	0.28	0.039
	343	7.11	0.29	0.040
	368	7.00	0.30	0.043
poly(MeEO$_2$GE)$_{2.5k}$	278	8.08	0.25	0.031
	300	8.00	0.27	0.034
	323	8.08	0.29	0.035
	343	8.01	0.30	0.038
	368	7.97	0.32	0.040
poly(EtEO$_2$GE)$_{2.5k}$	278	8.54	0.38	0.044
	300	8.54	0.39	0.045
	323	8.49	0.40	0.047
	343	8.56	0.40	0.047
	368	8.55	0.40	0.047

Table S4. Average numbers of hydrogen bonds within an intra-polymer calculated over 20 ns at 278K, 300K, 323K, 343K, and 368K.

Polymer	LCST (K)	Average Number of Intra-Polymers HBs in All O Atoms, (Per Oxygen in Polymer Residue)				
		**278 K**	**300 K**	**323 K**	**343 K**	**368 K**
poly(MeGE)	338.5	0.005	0.002	0.002	0.002	0.001
poly(EtGE)	283.3	0.004	0.002	0.002	0.002	0.002
poly(MeEOGE)	364.6	0.003	0.002	0.002	0.002	0.002
poly(EtEOGE)	314.3	0.003	0.003	0.003	0.002	0.002
poly(MeEO$_2$GE)	N.D.	0.002	0.002	0.002	0.002	0.002
poly(EtEO$_2$GE)	331.2	0.002	0.002	0.003	0.003	0.002

2. Additional Figures

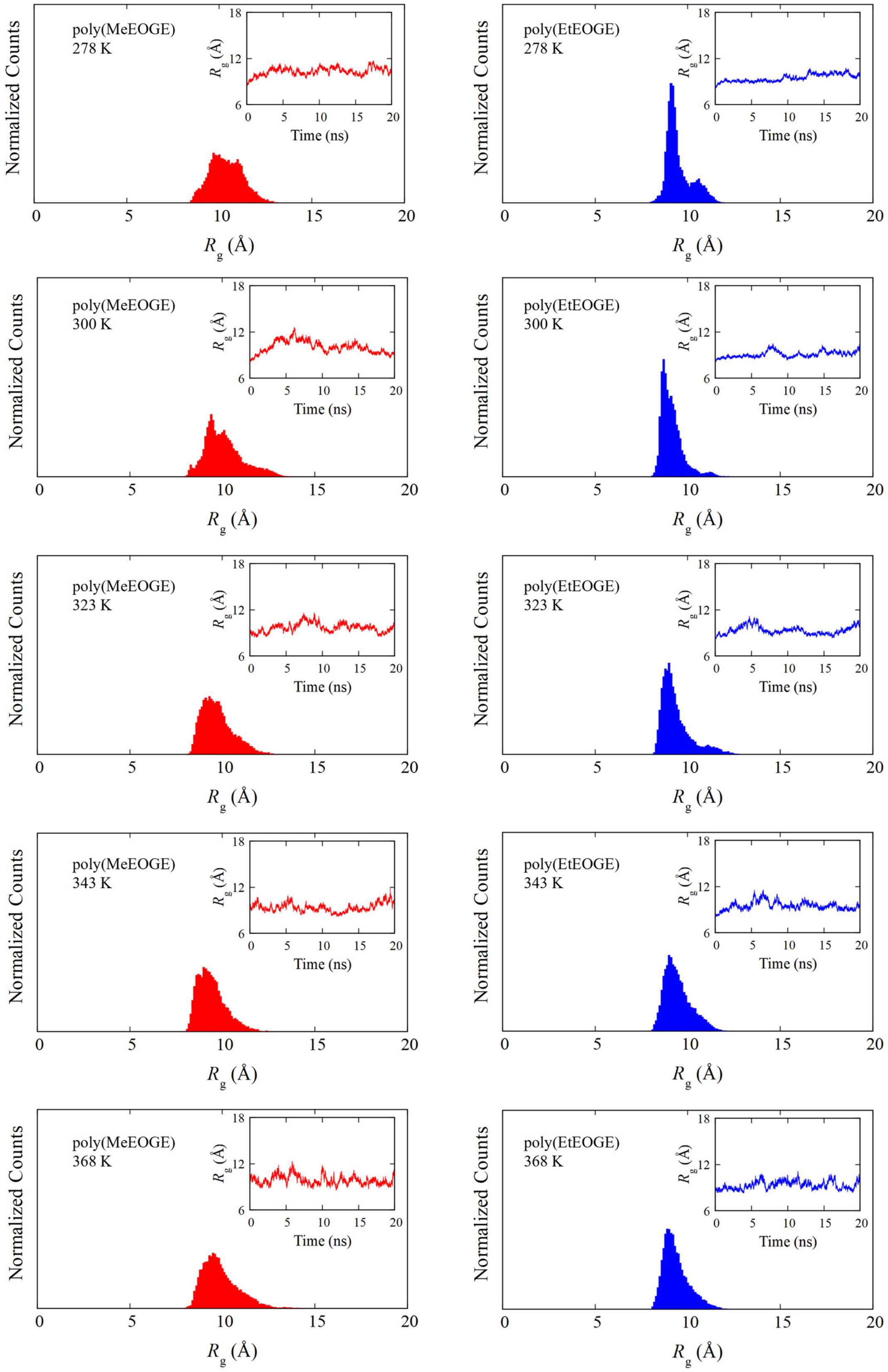

Figure S1. R_g distribution inserted for poly(MeEOGE)$_{2.5k}$ (left) and poly(EtEOGE)$_{2.5k}$ (right). The inset is the corresponding time dependence of R_g.

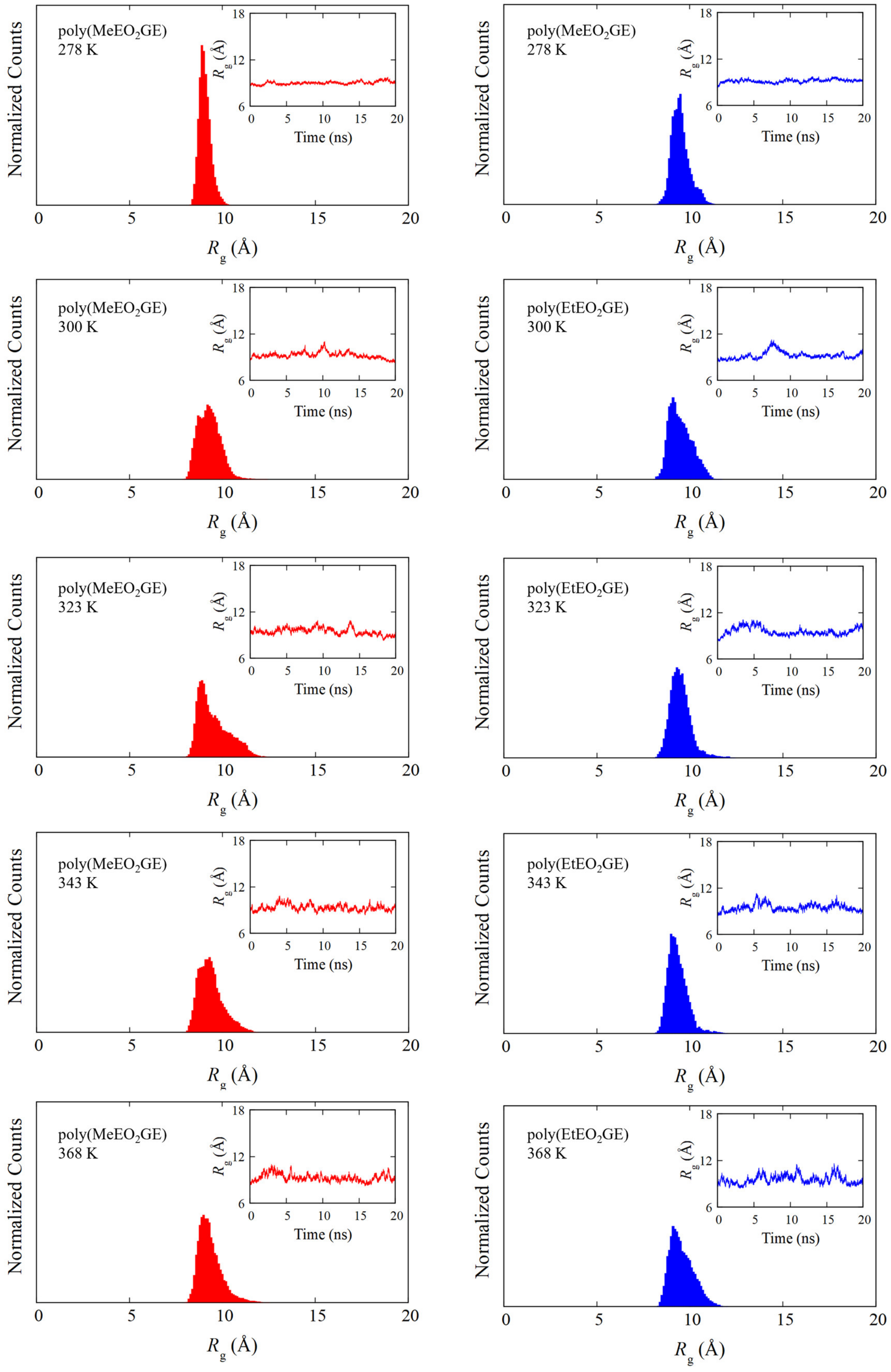

Figure S2. R_g distributions for poly($MeEO_2GE$)$_{2.5k}$ (left) and poly($EtEO_2GE$)$_{2.5k}$ (right). The inset is the corresponding time dependence of R_g.

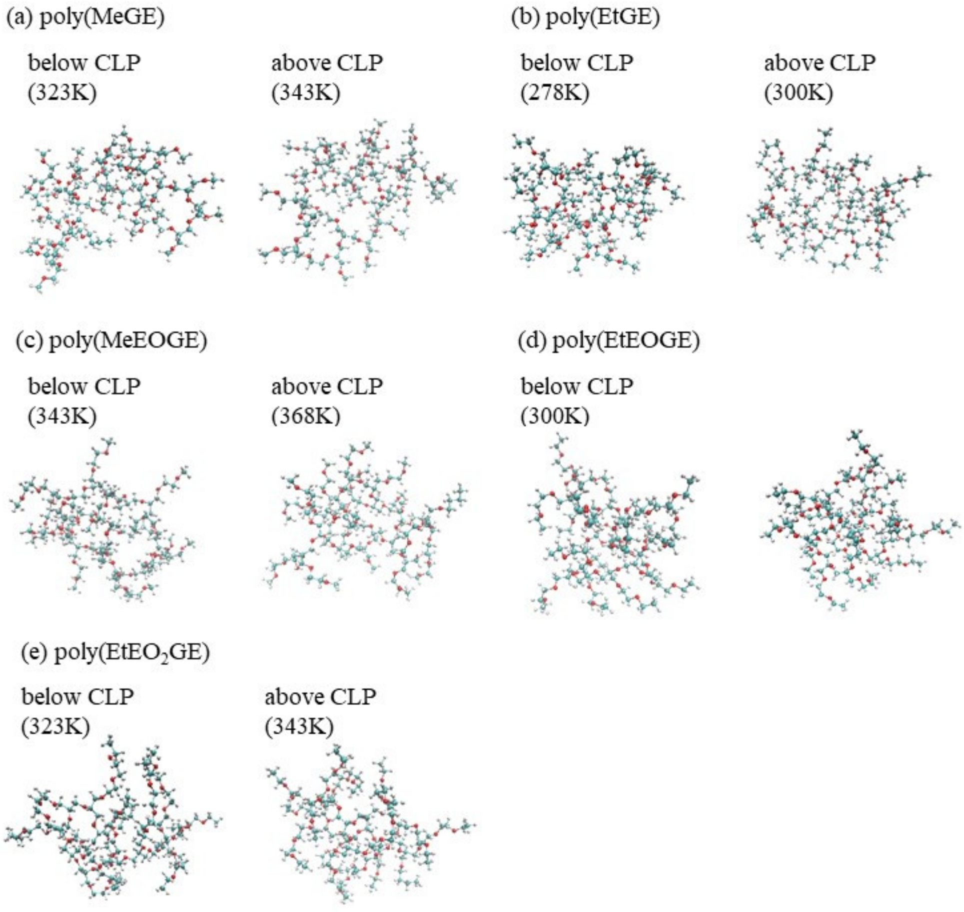

Figure S3. Snapshots of (a) poly(MeGE)$_{2.5k}$, (b) poly(EtGE)$_{2.5k}$, (c) poly(MeEOGE)$_{2.5k}$, (d) poly(EtEOGE)$_{2.5k}$ and (e) poly(EtEO$_2$GE)$_{2.5k}$ at the end of 20 ns below and above T_{CLP}. The carbon, oxygen, and hydrogen atoms of polymers are shown in green, red, and grey colors, respectively. Solvent water molecules have been omitted for clarity.

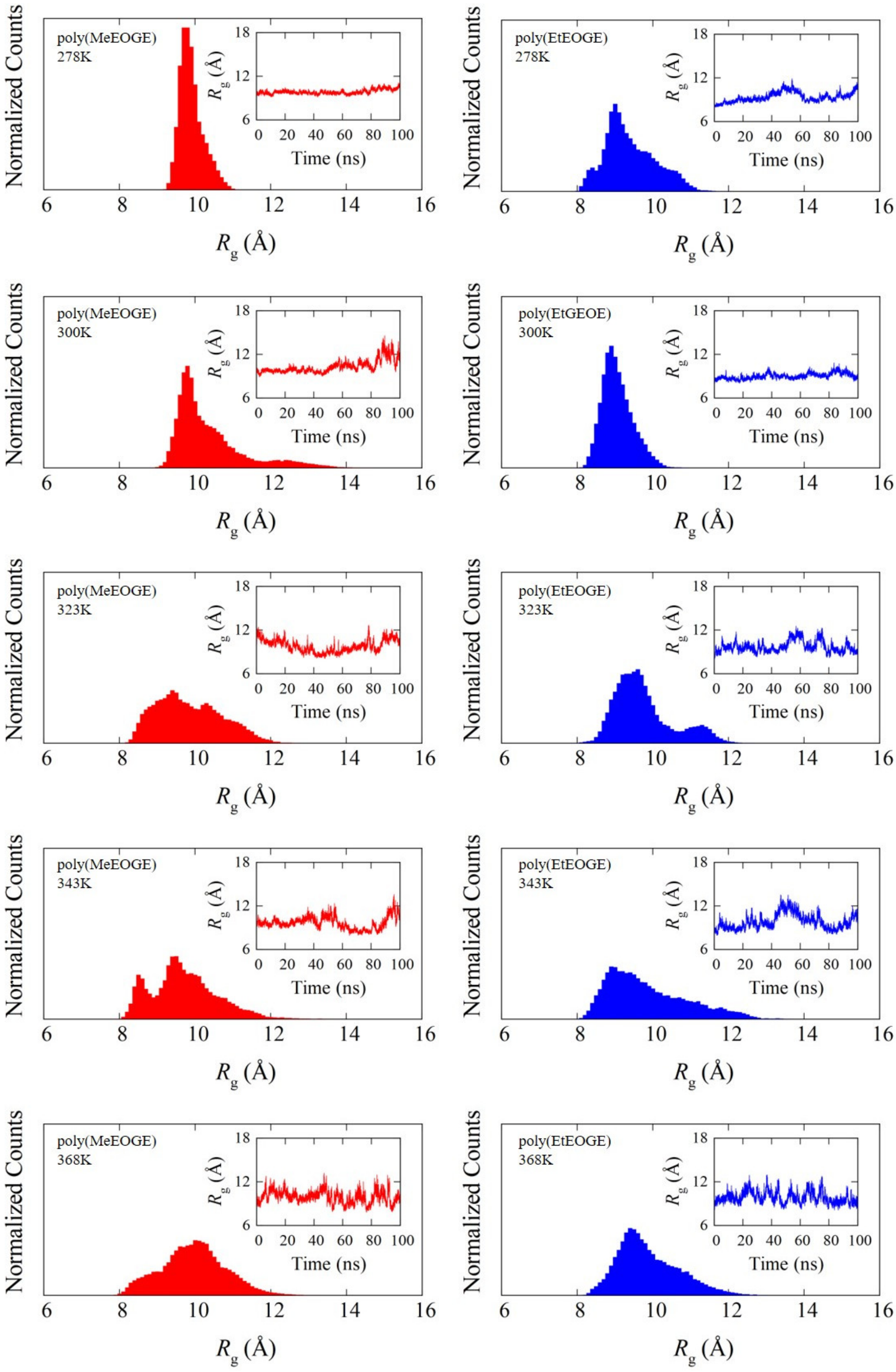

Figure S4. R_g distributions for poly(MeEOGE)$_{5k}$ (left) and poly(EtEOGE)$_{5k}$ (right). The inset is the corresponding time dependence of R_g.

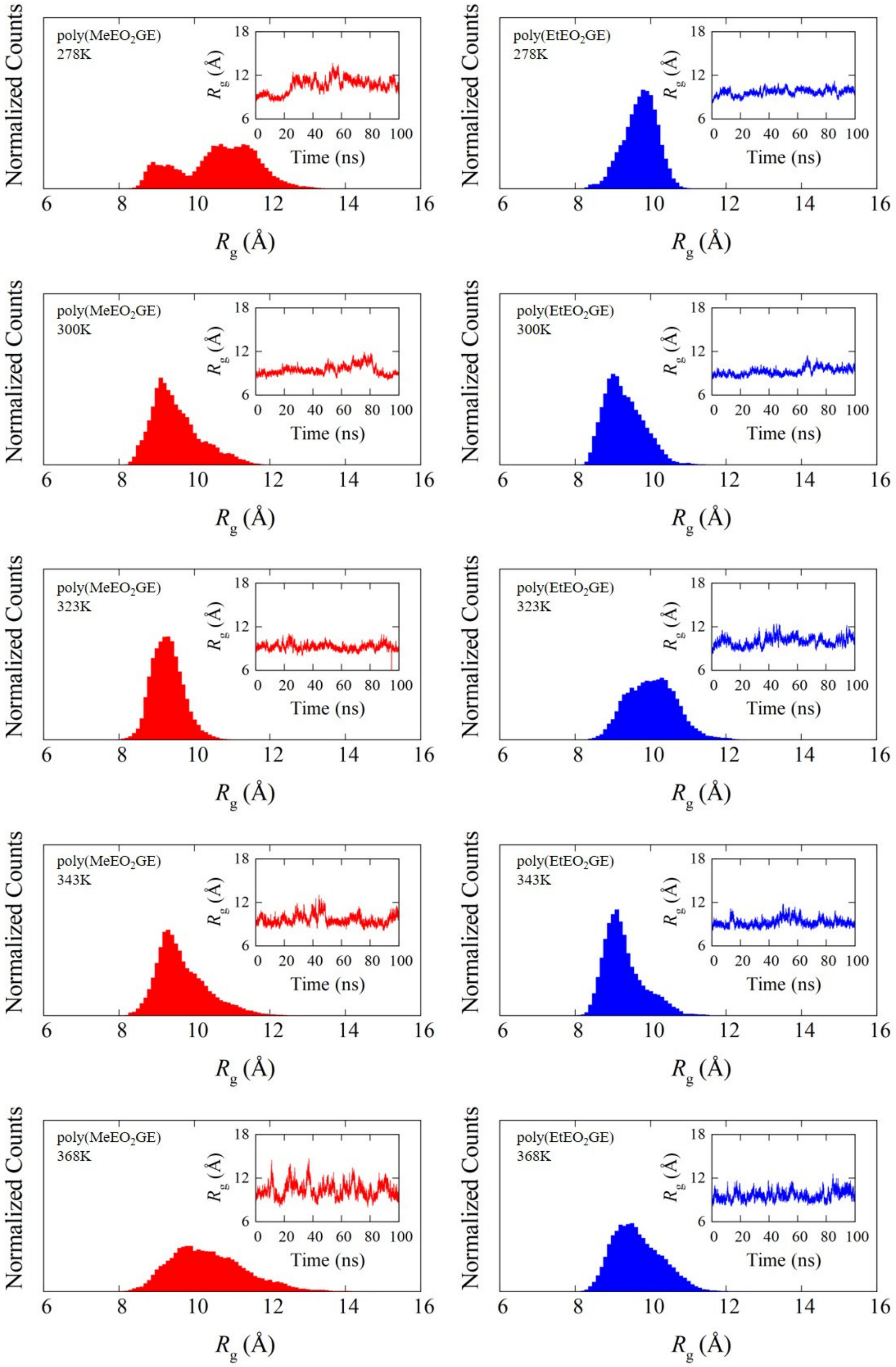

Figure S5. R_g distributions for poly(MeEO$_2$GE)$_{5k}$ (left) and poly(EtEO$_2$GE)$_{5k}$ (right). The inset is the corresponding time dependence of R_g.

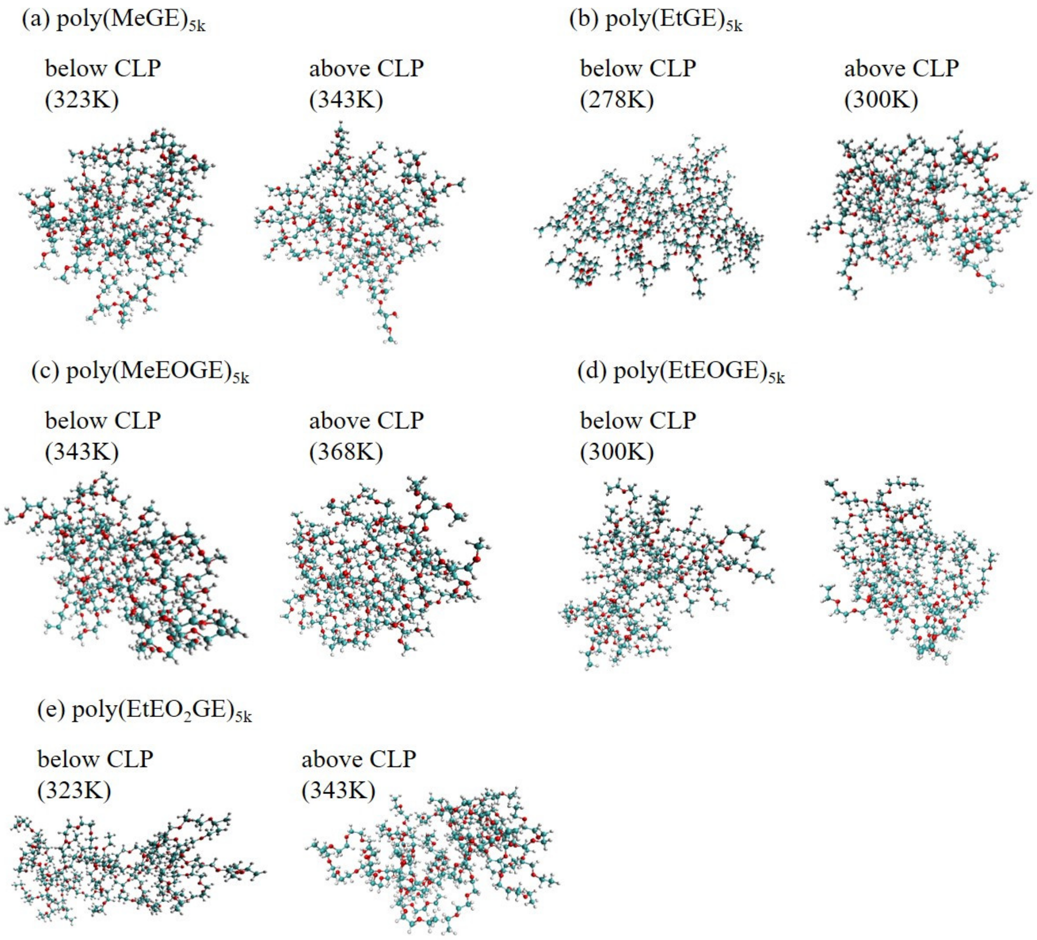

Figure S6. Snapshots of (a) poly(MeGE)$_{5k}$, (b) poly(EtGE)$_{5k}$, (c) poly(MeEOGE)$_{5k}$, (d) poly(EtEOGE)$_{5k}$ and (e) poly(EtEO$_2$GE)$_{5k}$ at the end of 20 ns below and above T_{CLP}. The carbon, oxygen, and hydrogen atoms of polymers are shown in green, red, and grey colors, respectively. Solvent water molecules have been omitted for clarity.

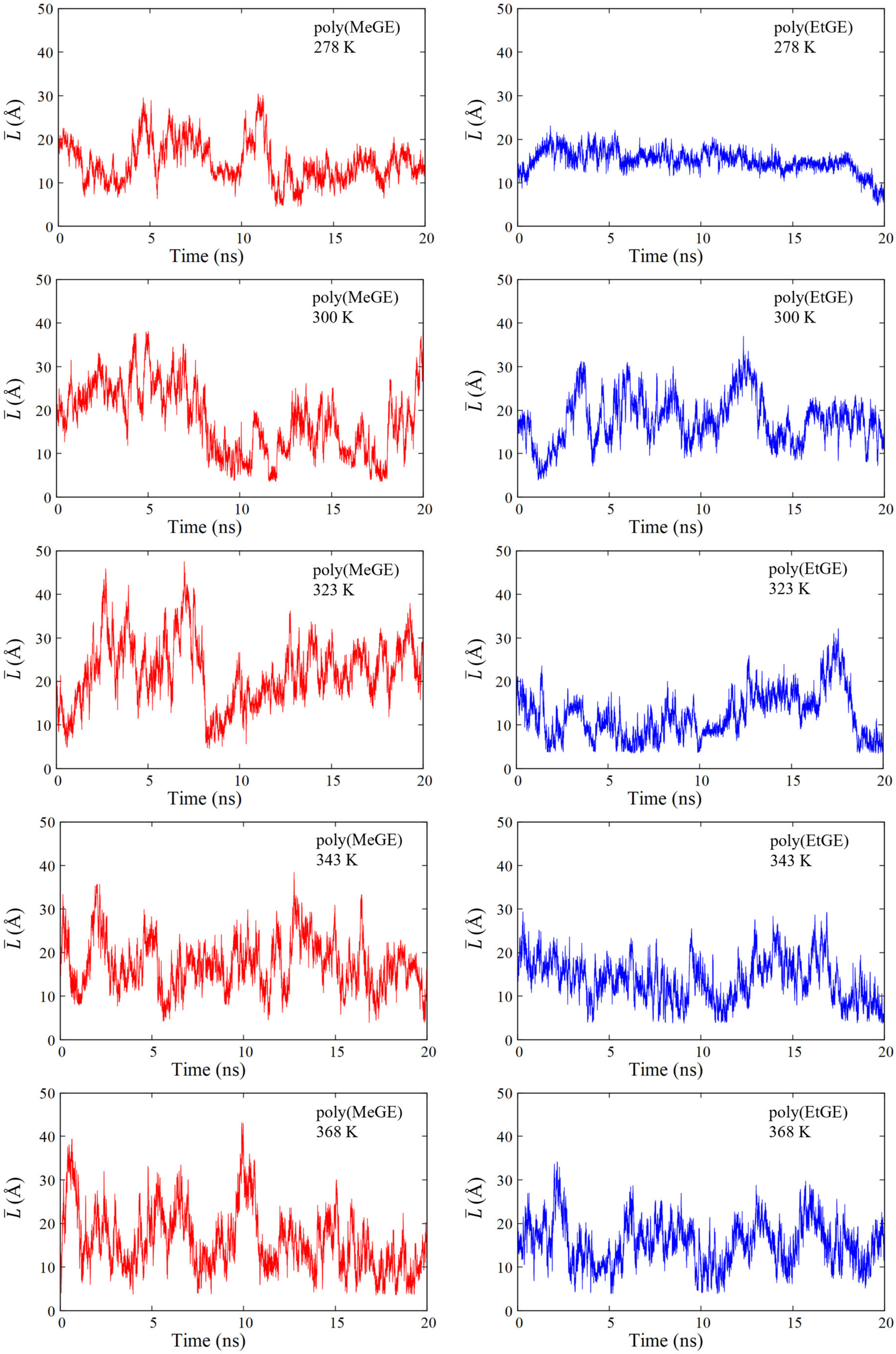

Figure S7. Time dependence of $\bar{L}$ for poly(MeGE)$_{2.5k}$ (left) and poly(EtGE)$_{2.5k}$ (right).

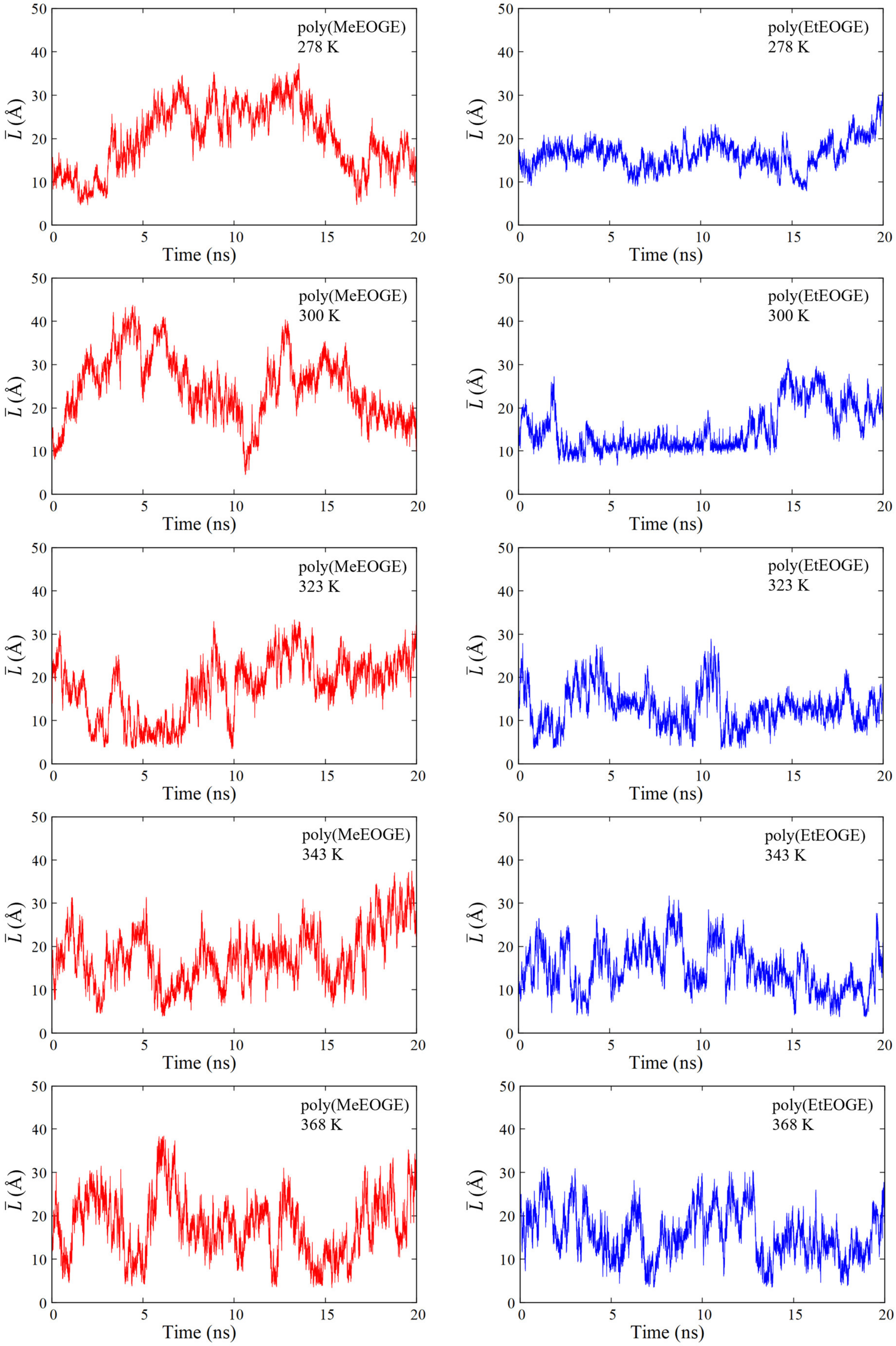

Figure S8. Time dependence of $\bar{L}$ for poly(MeEOGE)$_{2.5k}$ (left) and poly(EtEOGE)$_{2.5k}$ (right).

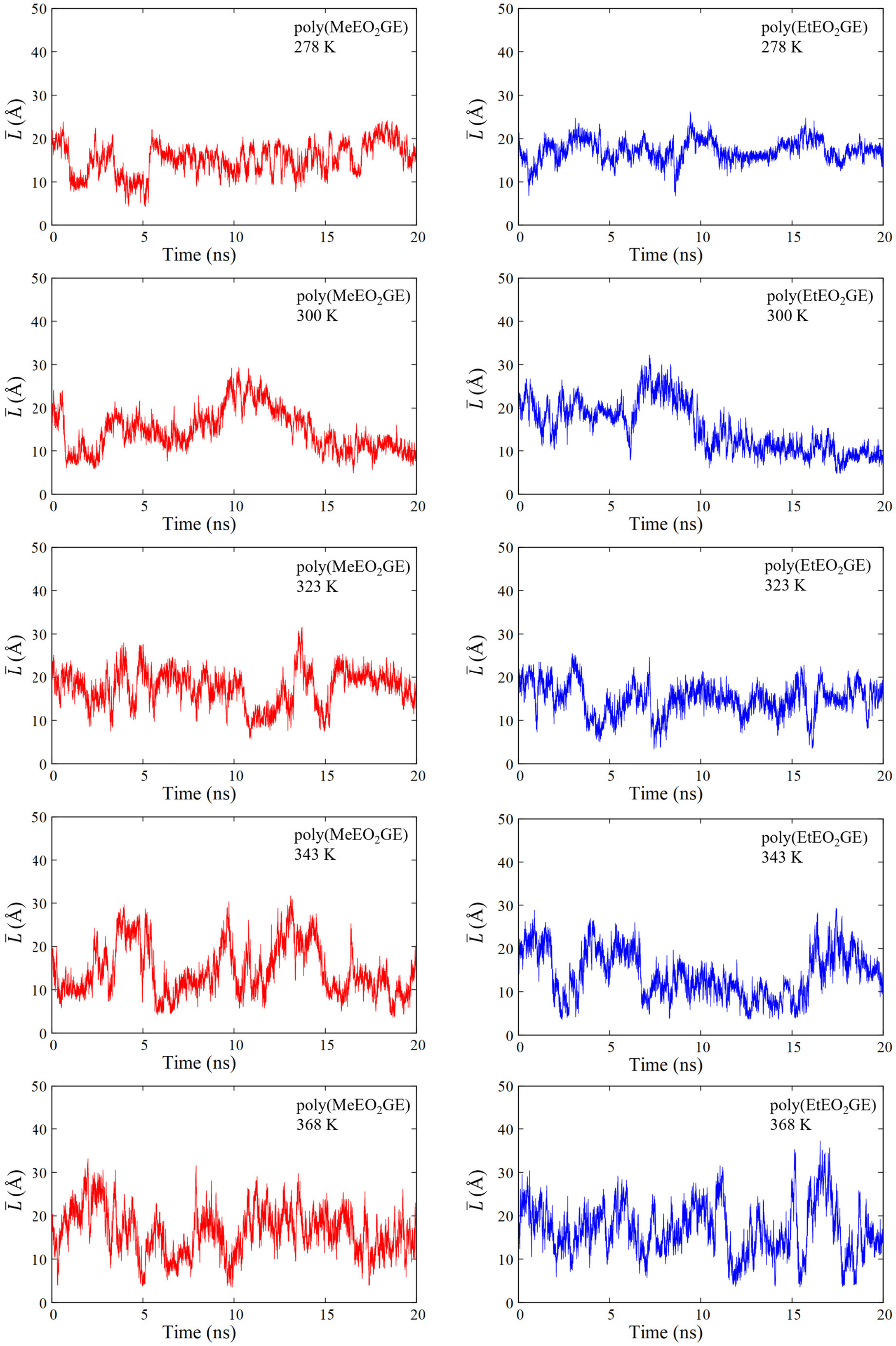

Figure S9. Time dependence of $\bar{L}$ for poly(MeEO2GE)$_{2.5k}$ (left) and poly(EtEO2GE)$_{2.5k}$ (right).

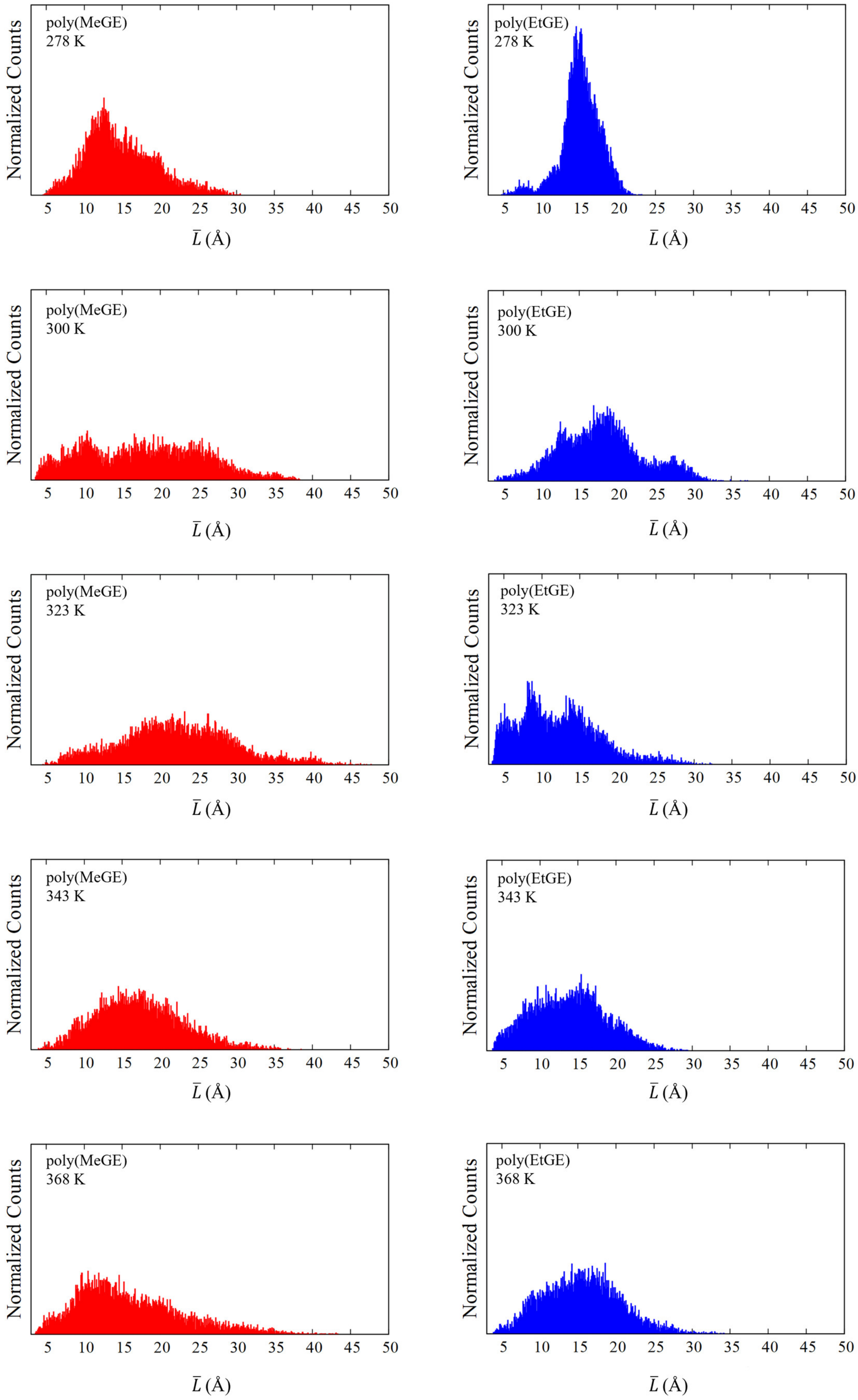

Figure S10. $\bar{L}$ distribution for poly(MeGE)$_{2.5k}$ (left) and poly(EtGE) (right)$_{2.5k}$.

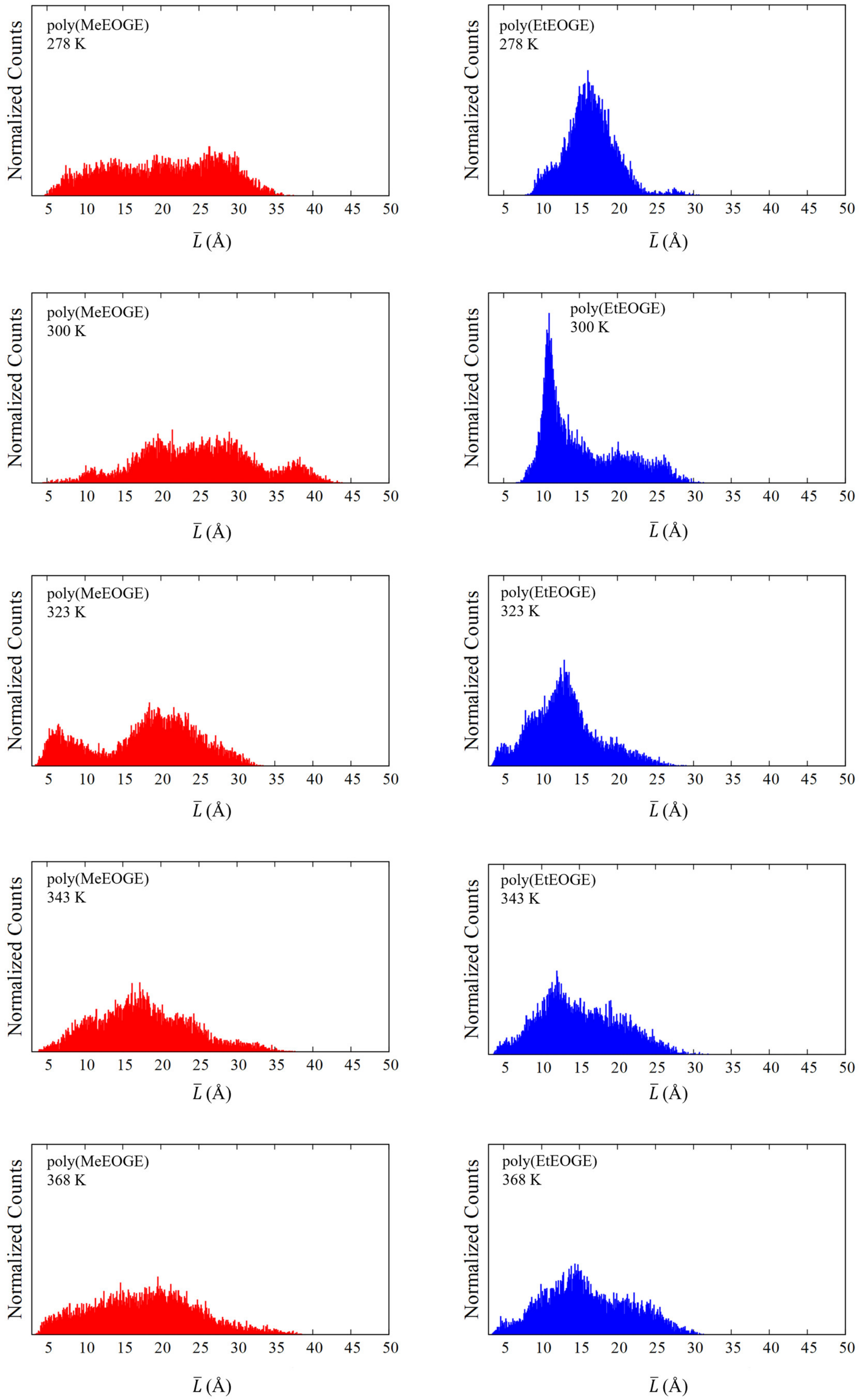

Figure S11. $\bar{L}$ distribution for poly(MeEOGE)$_{2.5k}$ (left) and poly(EtEOGE)$_{2.5k}$ (right).

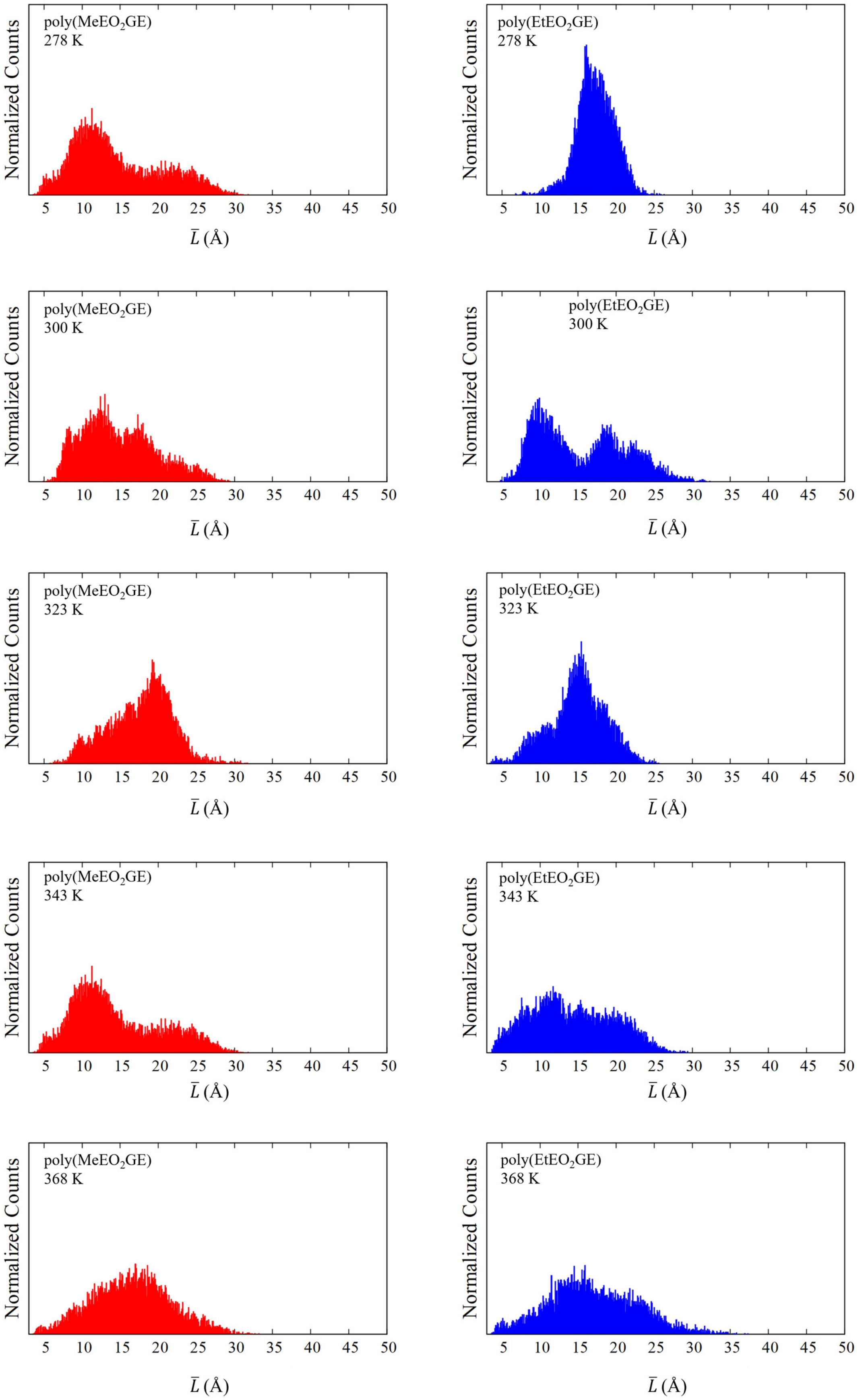

Figure S12. $\bar{L}$ distribution for poly(MeEO2GE)$_{2.5k}$ (left) and poly(EtEO2GE)$_{2.5k}$ (right).
